# C282Y Homozygosity Increases Erythrocyte Turnover and Decreases HbA1c—A Population-Based Study

**DOI:** 10.3390/ijms27052410

**Published:** 2026-03-05

**Authors:** Rebekka Hillingsø, Alisa Devedzic Kjaergaard, Morten Kranker Larsen, Thomas Mandrup-Poulsen, Henrik Enghusen Poulsen, Mathis Mottelson, Jesper Brix Petersen, Børge Grønne Nordestgaard, Hans Carl Hasselbalch, Stig Egil Bojesen, Jens Helby, Andreas Glenthøj, Christina Ellervik

**Affiliations:** 1 Faculty of Health and Medical Sciences, University of Copenhagen, 2200 Copenhagen, Denmark; rebekkahillingsoe@gmail.com; 2 Department of Laboratory Medicine, Boston Children’s Hospital, Boston, MA 02115, USA; 3 Department of Psychiatry, Horsens Hospital, 8700 Horsens, Denmark; 4 Department of Hematology, Zealand University Hospital, 4000 Roskilde, Denmark; mokl@regionsjaelland.dk (M.K.L.); hkhl@regionsjaelland.dk (H.C.H.); 5 Department of Biomedical Sciences, Faculty of Health and Medical Sciences, University of Copenhagen, 2200 Copenhagen, Denmark; tmpo@sund.ku.dk; 6 Department of Clinical Medicine, Faculty of Health and Medical Sciences, University of Copenhagen, 2200 Copenhagen, Denmark; henrik.enghusen.poulsen.01@regionh.dk (H.E.P.); boerge.nordestgaard@regionh.dk (B.G.N.); stig.egil.bojesen@regionh.dk (S.E.B.); andreas.glenthoej@regionh.dk (A.G.); 7 Department of Endocrinology, Copenhagen University Hospital-Bispebjerg and Frederiksberg, Bispebjerg, 2400 Copenhagen, Denmark; 8 Department of Hematology, Danish Red Blood Cell Center, Rigshospitalet, 2100 Copenhagen, Denmark; jesper.petersen@regionh.dk; 9 Department of Clinical Biochemistry, Copenhagen University Hospital-Herlev and Gentofte, 2730 Herlev, Denmark; 10 The Copenhagen General Population Study, Copenhagen University Hospital-Herlev and Gentofte, 2730 Herlev, Denmark; 11 The Copenhagen City Heart Study, Copenhagen University Hospital-Bispebjerg and Frederiksberg, 2730 Herlev, Denmark; 12 Department of Pathology, Harvard Medical School, Boston, MA 02115, USA; 13 Department of Clinical Biochemistry, Zealand University Hospital, 4600 Køge, Denmark

**Keywords:** HFE, C282Y/C282Y, HbA1c, erythrocytes, red blood cells, reticulocytes, erythrocyte turnover, erythrocyte survival, transferrin saturation, oxidative stress, MCHC, MCV

## Abstract

Individuals with C282Y/C282Y in the hemochromatosis *HFE* gene have increased iron levels, which catalyze the formation of reactive oxygen species, and an increased risk of diabetes. These individuals may have disproportionately lower hemoglobin A1c (HbA1c) due to increased erythrocyte turnover, decreased erythrocyte counts, and/or an increased mean corpuscular hemoglobin concentration (MCHC). In the Copenhagen General Population Study (N = 103,734) and the Danish General Suburban Population Study (GESUS, N = 20,003), we investigated the association between C282Y/C282Y (N = 399) and other *HFE* genotypes with erythrocyte count, MCHC, mean corpuscular volume (MCV), red cell distribution width (RDW), and high-sensitivity C-reactive protein (hsCRP). In GESUS, we additionally investigated the association with oxidative stress (by 8-oxo-7,8-dihydroguanosine and 8-oxo-7,8-dihydro-2′-deoxyguanosine), reticulocyte count, reticulocyte hemoglobin, reticulocyte percentage as a proxy for erythrocyte turnover, and HbA1c in linear regressions adjusted for age, sex, cohort, and blood donation. We investigated the mediation between *HFE* genotype and HbA1c. Compared to non-carriers, individuals with C282Y/C282Y had increased p-iron, transferrin saturation, ferritin, hsCRP, oxidative stress, reticulocyte counts, reticulocyte percentage (1.24% vs. 1.06%, *p* = 1.7 × 10^−5^) as a proxy for erythrocyte turnover, MCHC (344 vs. 340 g/L, *p* = 1.7 × 10^−12^), MCH, MCV, reticulocyte hemoglobin, p-glucose (5.6 vs. 5.4, *p* = 0.007), bilirubin, and LDH and decreased RDW, erythrocyte counts (4.49 × 10^12^/L vs. 4.61 × 10^12^/L, *p* = 6.1 × 10^−11^), estimated erythrocyte survival, and HbA1c (36 vs. 38 mmol/mol, *p* = 0.01). The associations were similar, although attenuated, for other *HFE* genotypes. The association between the *HFE* genotype and decreased HbA1c was partially mediated by increased transferrin saturation, MCHC, MCV, and decreased erythrocyte count, but not by hsCRP, reticulocyte count, oxidative stress, or blood donation. In conclusion, while C282Y/C282Y and other *HFE* genotypes increased erythrocyte turnover, the disproportionately decreased HbA1c level was explained by fewer but larger erythrocytes filled with more hemoglobin and removed earlier from circulation, thus diluting the relative concentration of intracellular glucose per hemoglobin molecule.

## 1. Introduction

Hereditary hemochromatosis (HH) is a genetic iron overload disorder. It is caused by homozygosity for the functional missense mutation C282Y (rs1800562[G→A]) in the *HFE* gene in more than 80% of hemochromatosis patients in populations of Northern European descent [1]. The frequency of the A-allele ranges from 5 to 10% in Northern European populations [2], and the prevalence of homozygosity for C282Y (C282Y/C282Y) ranges from 0.30 to 0.60% in similar populations [3,4,5,6,7]. The HFE protein is primarily expressed in hepatocytes, where it helps regulate hepcidin production in response to body iron stores. The C282Y mutation disrupts a disulfide bond in the HFE protein, which is essential for the function of the protein. In *HFE* hemochromatosis (C282Y/C282Y), mutant HFE disrupts this signaling, leading to inappropriately low hepcidin levels and uncontrolled iron absorption via ferroportin on enterocytes, reflected by a high serum iron concentration, ferritin concentration, and transferrin saturation (TSAT) [8,9]. The long-term accumulation of iron is mainly in the liver but also in endocrine organs and joints [8,9]. We have previously shown that C282Y/C282Y is associated with increased risk of liver disease and diabetes and increased mortality in individuals with diabetes in Danish population cohorts [3,10,11]. Another functional but less pathogenic variant (H63D, rs1799945[C→G]) that confers the partial loss of the HFE protein may also cause iron overload, particularly as a compound heterozygote with C282Y [8].

In multiple genome-wide association studies (GWASs), C282Y is not only associated with elevated serum iron, ferritin, and TSAT [12,13,14] but also with elevated hemoglobin and elevated erythrocyte indices such as mean cell volume (MCV), hematocrit, mean corpuscular hemoglobin (MCH), and mean corpuscular hemoglobin concentration (MCHC) [15,16]. These observations lead to the expectation that the erythrocyte counts would also be higher with C282Y, but the contrasting finding of decreased erythrocyte counts has not been mentioned [15,16]. This prompted us to investigate this further.

Iron overload causes the formation of reactive oxygen species (ROS) [17], which is also associated with the deformation of erythrocytes, which lose their biconcave shape and become more rigid [18]. We previously showed that individuals carrying C282Y had increased 8-oxo-7,8-dihydroguanosine (8-oxoGuo) [17], a biomarker of oxidative stress and associated with diabetes [17], compared to non-carriers. Peripheral blood smears in individuals with *HFE* variants suggest an abnormal erythrocyte morphology [19,20]. Several functional studies show that the change in erythrocyte morphology is moderately reversible in well-regulated *HFE* hemochromatosis patients treated with phlebotomy [21,22]. In individuals with C282Y/C282Y, these morphologic and functional changes may make the erythrocytes more prone to early ageing and premature removal from the circulation, which would explain the finding of decreased erythrocyte counts found in GWASs, with an increased compensatory production of reticulocytes, a process called erythrocyte turnover.

Hemoglobin A1c (HbA1c) is the proportion of hemoglobin that is non-enzymatically glycated and is an indirect measure of long-term glucose exposure over the lifespan of erythrocytes. Conditions that affect hemoglobin or erythrocyte turnover will affect the accuracy of HbA1c [23,24]. It has previously been shown that HbA1c decreases with increasing erythrocyte turnover [25], MCHC, and MCV [26]. Understanding the mechanisms by which *HFE* affects HbA1c is clinically relevant because HbA1c is widely used for the diagnosis and monitoring of diabetes, and its misinterpretation in C282Y/C282Y carriers could lead to misdiagnosis. We therefore hypothesized that C282Y/C282Y and, to a lesser extent, other *HFE* genotypes are associated with reduced HbA1c due to increased erythrocyte turnover with proportionately more reticulocytes with less hemoglobin available for glycosylation and/or by an increased hemoglobin concentration in larger erythrocytes (MCHC and MCV), causing the dilution of the relative concentration of intracellular glucose per hemoglobin molecule.

In the Copenhagen General Population Study (CGPS) and the Danish General Suburban Population Study (GESUS), we investigated the association between *HFE* genotypes, erythrocyte turnover, and HbA1c and whether the association with HbA1c was mediated by transferrin saturation, MCHC, MCV, oxidative stress, hsCRP, erythrocyte count, reticulocyte count, or blood donation. This has, to our knowledge, not been investigated before.

## 2. Results

Baseline characteristics of 123,737 individuals in CGPS and GESUS stratified by HFE genotype are presented in Table 1. The distribution of genotypes was similar in CGPS and GESUS. The frequency was 5.9% for the A-allele (rs1800562) for C282Y and 13.1% for the G-allele (rs1799945) for H63D (Supplementary Table S1), and the prevalence of C282Y/C282Y (N = 399) was 0.32% in the two cohorts combined (Table 1). Individuals with C282Y/C282Y had increased plasma iron concentration, transferrin saturation, ferritin concentration, hematocrit, hemoglobin, and MCV, but decreased red cell distribution width (RDW), and there was a trend across the *HFE* genotypes (H63D/NC, C282Y/NC, H63D/H63D, C282Y/H63D, and C282Y/C282Y). Individuals with C282Y/C282Y and C282Y/H63D had increased 8-oxoGuo, C282Y/C282Y had increased hsCRP, and all genotypes had increased ALAT compared to non-carriers (Table 1). The characteristics were similar for both cohorts (Supplementary Tables S2 and S3).

### 2.1. Erythrocytes and Reticulocytes

**Figure 1:**
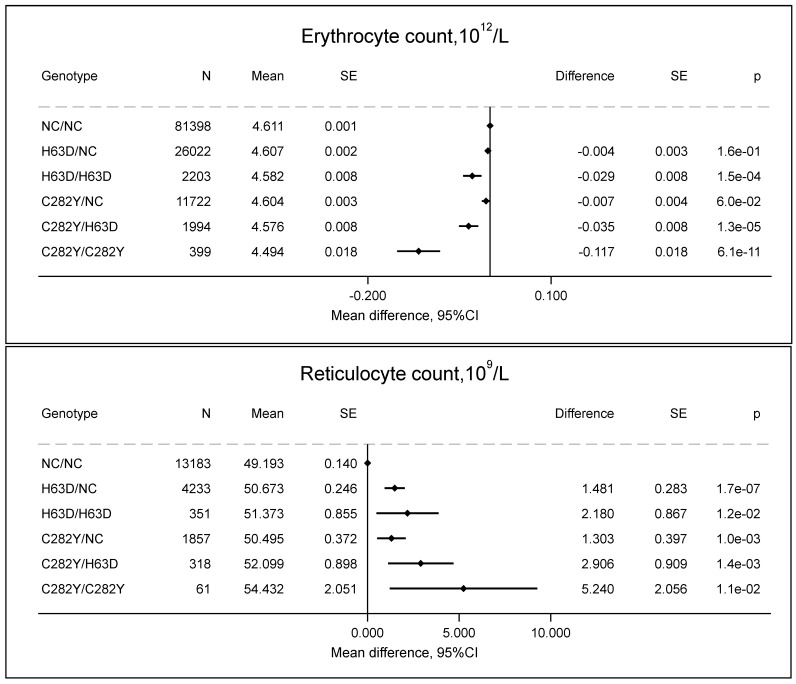
Erythrocyte and reticulocyte count by *HFE* genotypes compared to non-carrier/non-carrier (NC/NC). Values are predicted means with standard error (SE), and predicted mean differences with SE from a linear regression. e is scientific notation for “10 to the power of”.

**Figure 2:**
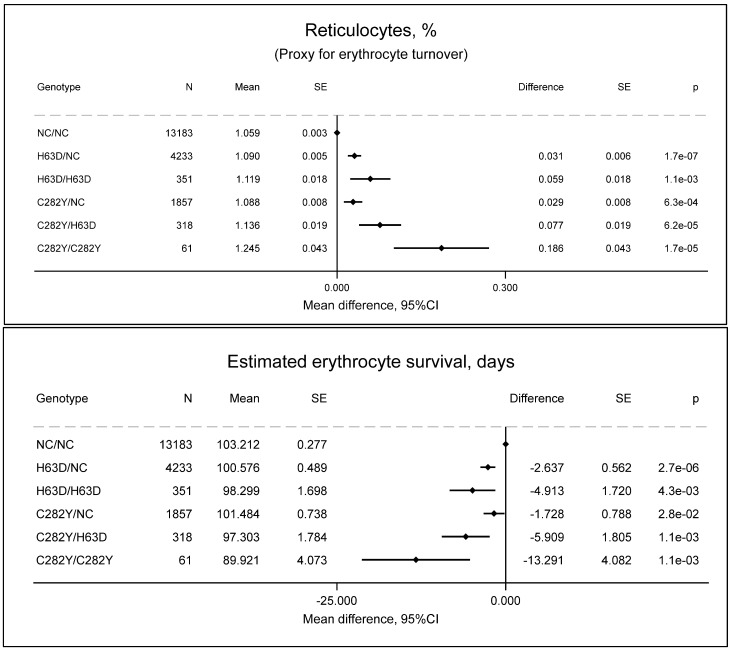
Estimated erythrocyte turnover and survival by *HFE* genotypes compared to non-carrier/non-carrier (NC/NC). Values are predicted means with standard error (SE) and predicted mean differences with SE from a linear regression. e is scientific notation for “10 to the power of”.

Individuals with C282Y/C282Y had increased reticulocyte counts (54 × 10^9^/L vs. 49 × 10^9^/L, *p* = 0.01) (Figure 1), increased reticulocyte percentage (1.24% vs. 1.06%, *p* = 1.7 × 10^−5^) as a proxy for erythrocyte turnover (Figure 2), and decreased erythrocyte counts (4.49 × 10^12^/L vs. 4.61 × 10^12^/L, *p* = 6.1 × 10^−11^) (Figure 1) compared to non-carriers. Assuming that the erythrocyte turnover lasts approximately 1 day and that the erythrocyte turnover equals the erythrocyte removal, the estimated erythrocyte survival in individuals with C282Y/C282Y is 90 days vs. 103 days (*p* = 0.001) compared to non-carriers (Figure 2); while the exact values of erythrocyte survival are uncertain, the erythrocyte survival in individuals with C282Y/C282Y is decreased.

### 2.2. Hemoglobin Concentration in Erythrocytes and Reticulocytes

**Figure 3:**
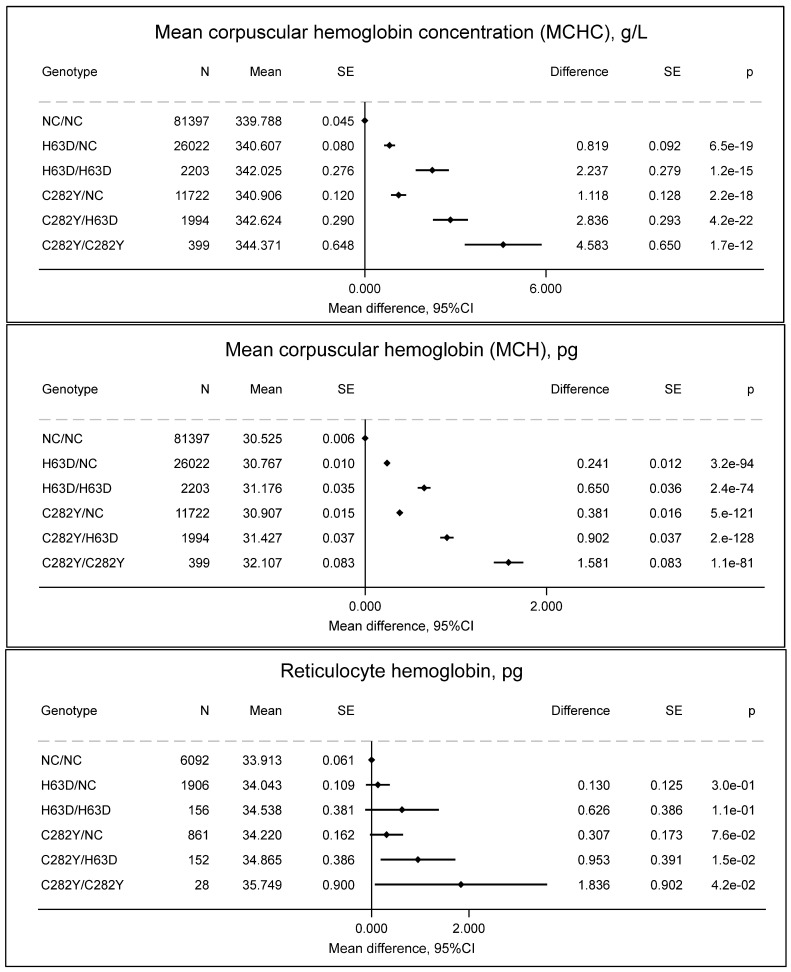
Mean corpuscular hemoglobin concentration (MCHC, g/L), mean corpuscular hemoglobin (pg), and reticulocyte hemoglobin (pg) by *HFE* genotypes compared to non-carrier/non-carrier (NC/NC). Values are predicted means with standard error (SE) and predicted mean differences with SE from a linear regression. e is scientific notation for “10 to the power of”.

Individuals with C282Y/C282Y had increased MCHC (344 vs. 340 g/L, *p* = 1.7 × 10^−12^), MCH (32.1. vs. 30.5 pg, *p* = 1.1 × 10^−81^), and reticulocyte hemoglobin (35.7 vs. 33.9 pg, *p* = 0.04) (Figure 3), which indicate differences in erythropoiesis.

### 2.3. Other Biomarkers

**Figure 4:**
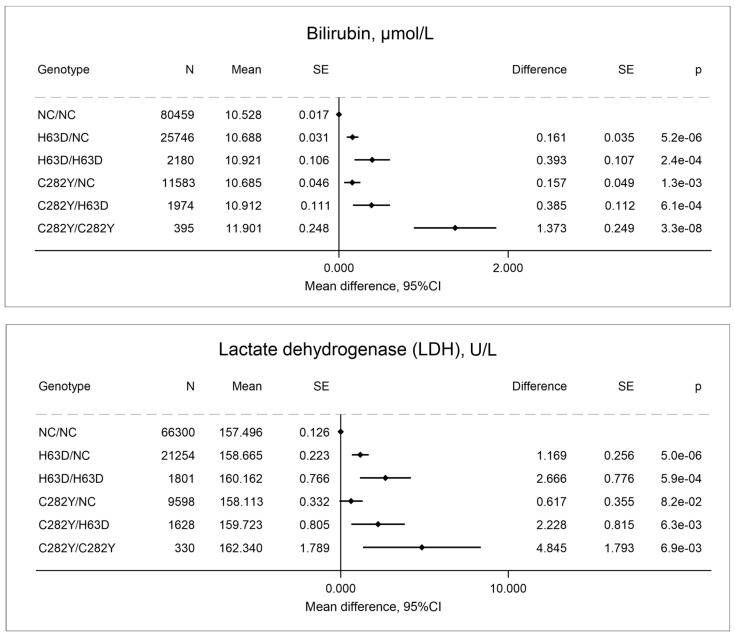
Bilirubin and lactate dehydrogenase (LDH) by *HFE* genotypes compared to non-carrier/non-carrier (NC/NC). Values are predicted means with standard error (SE) and predicted mean differences with SE from a linear regression. e is scientific notation for “10 to the power of”.

Other indirect biomarkers of increased erythrocyte turnover or liver damage in individuals with C282Y/C282Y included elevated bilirubin and LDH (Figure 4). The elevation in bilirubin and LDH in individuals with C282Y/C282Y is consistent with increased erythrocyte destruction, liver damage (also evidenced by elevated ALAT, Table 1), or both.

### 2.4. HbA1c and Plasma Glucose in the GESUS Cohort

**Figure 5:**
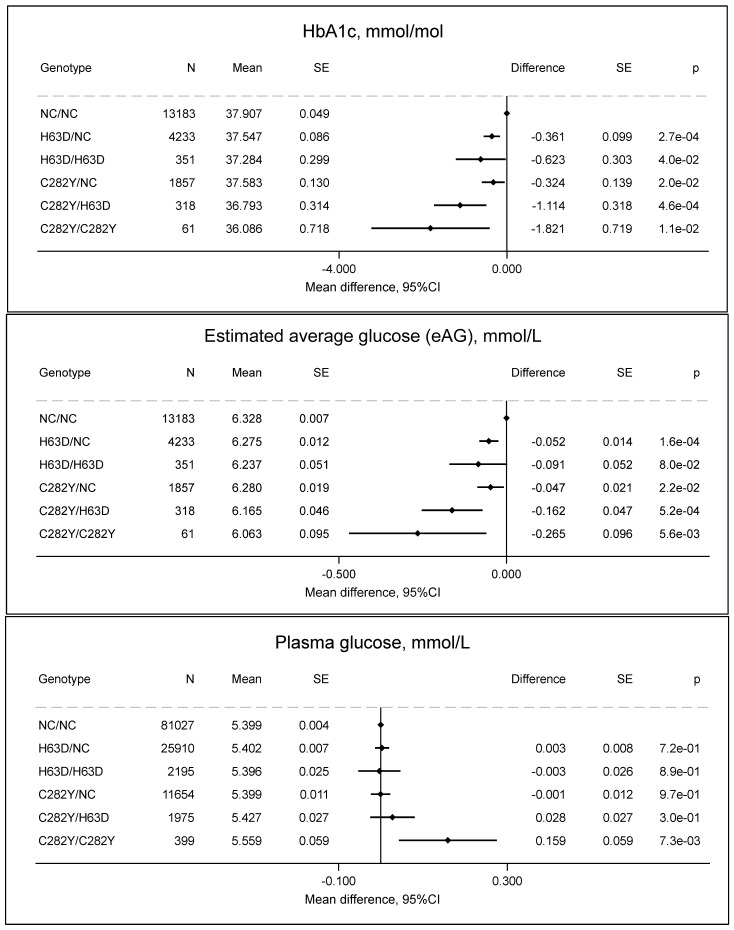
HbA1c (mmol/mol), estimated average glucose (eAG), and p-glucose by *HFE* genotypes compared to non-carrier/non-carrier (NC/NC). Values are predicted means with standard error (SE) and predicted mean differences with SE from a linear regression. e is scientific notation for “10 to the power of”.

HbA1c was 2 mmol/mol lower in C282Y/C282Y (36 mmol/mol) than in non-carriers (38 mmol/mol, *p* = 0.01) (Figure 5), and estimated average glucose showed a corresponding pattern. Plasma glucose was 0.16 mmol/L higher in individuals with C282Y/C282Y compared to non-carriers (*p* = 0.007). The discordant association between reduced HbA1c and elevated plasma glucose in individuals with C282Y/C282Y is particularly noteworthy and suggests that HbA1c may not be a reliable biomarker in this population.

### 2.5. Other HFE Genotypes

Other *HFE* genotypes showed a similar but attenuated pattern, such that the differences for C282Y/H63D and H63D/H63D were approximately 30–50% of that for C282Y/C282Y, and the differences for C282Y/NC and H63D/NC were approximately 10–25% of that for C282/C282Y, although 95% CIs were overlapping in some analyses, indicating less precision. Furthermore, only C282Y/C282Y, and not the other *HFE* genotypes, was associated with increased p-glucose.

### 2.6. Sensitivity Analyses

Across all regression models, there was no interaction (*p* > 0.05) between *HFE* genotypes and sex, indicating that the effect of *HFE* genotypes on the biomarkers did not differ between women and men. In fully adjusted models, results were similar across all genotypes (Supplementary Table S4).

### 2.7. Mediation Analyses

**Figure 6:**
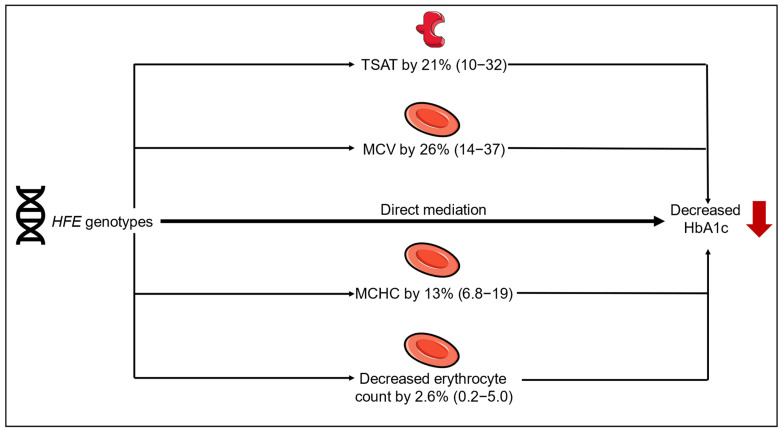
Mediation analysis by *HFE* genotypes and decreased HbA1c. The figure was partly generated using pictures from Servier Medical Art, provided by Servier, licensed under a Creative Commons Attribution 3.0 Unported license.

The association between *HFE* genotypes on decreased HbA1c was partly mediated by increased transferrin saturation, MCHC, and MCV, and decreased erythrocyte count, but not by hsCRP, reticulocyte count, oxidative stress, or blood donation (Figure 6). Although we expected that a higher reticulocyte count (young erythrocytes with lower hemoglobin glycosylation) would mediate the association, mediation analyses did not confirm this hypothesis.

## 3. Discussion

In this study, individuals with C282Y/C282Y had higher reticulocyte counts and higher estimated erythrocyte turnover based on the reticulocyte percentage, together with lower erythrocyte counts, shorter estimated erythrocyte survival, and lower HbA1c compared to non-carriers. Other *HFE* genotypes showed similar but attenuated associations. In contrast, p-glucose was increased in individuals with C282Y/C282Y. The mediation analyses suggested that it was not the erythrocyte turnover *per se* that mediated the association between *HFE* genotype and decreased HbA1c, but rather the increased transferrin saturation and the increased MCHC within fewer but larger erythrocytes, which rather reflects differences in erythropoiesis and erythrocyte removal. This was also supported by the finding that individuals with C282Y/C282Y had increased reticulocyte hemoglobin, indicating late erythroid maturation in the bone marrow before the release of reticulocytes into the peripheral blood.

Erythrocyte turnover is a continuous physiological process involving the removal of senescent erythrocytes and the replacement of the erythrocyte pool with newly produced reticulocytes. Erythrocyte clearance occurs mainly in the spleen and liver via macrophages, whereby hemoglobin is degraded into heme and globin. Heme is subsequently metabolized to bilirubin, while iron is recycled to the bone marrow for erythropoiesis. In this study, bilirubin and LDH were higher in individuals with C282Y/C282Y compared to non-carriers, which may reflect increased erythrocyte destruction in the liver or spleen, liver damage (increased ALAT) due to iron accumulation, or both.

Although estimated erythrocyte turnover was higher in individuals with C282Y/C282Y compared to non-carriers, erythrocyte counts were lower, indicating that the circulating erythrocyte pool was maintained at a lower set point. This was also reflected by a decreased RDW in individuals with C282Y/C282Y indicating a more homogenous pool of younger erythrocytes. Nonetheless, the hemoglobin concentration was maintained within the normal range and was, on average, even higher in C282Y/C282Y compared to non-carriers, indicating preserved erythropoietic compensation. The increased hemoglobin concentration packed in fewer erythrocytes is consistent with increased MCH, accompanied by increased MCV, and MCHC, suggesting larger and younger erythrocytes with increased hemoglobin content and concentration. Although reticulocytes have a negligible amount of glycated hemoglobin [27,28], it was not the increased reticulocyte count *per se* that mediated the association between *HFE* genotype and decreased HbA1c in this study. Whether these hematological features reflect compensatory responses to increased erythrocyte clearance, increased iron availability from both intestinal absorption or reticuloendothelial iron mobilization, or a combination of these mechanisms is uncertain. However, in addition to increased intestinal iron absorption, reduced hepcidin expression in C282Y/C282Y is associated with enhanced ferroportin-mediated iron export from reticuloendothelial macrophages, increasing the availability of recycled iron for erythropoiesis [8].

Two key features of programmed erythrocyte removal (eryptosis) are erythrocyte shrinkage and membrane phospholipid scrambling driven by Ca^2+^ influx, which causes phosphatidylserine (PS) to move from the inside to the surface of the erythrocytes [29]. This process can be triggered by oxidative stress [30] and could be a likely mechanism for the decreased erythrocyte count in individuals with C282Y/C282Y and to a lesser extent in other *HFE* genotypes. Thus, unliganded iron in individuals with C282Y/C282Y, and, to a lesser extent, other *HFE* genotypes, participates in the Fenton reaction, creating reactive oxygen species, which we have previously shown [17,31]. Increased oxidative stress is also associated with a rigid erythrocytic membrane [21,22,32]. And finally, phosphatidylserine expression on the surface of erythrocytes has been observed across all *HFE* mutations [29].

Evidence from other studies supports morphological [19,20] and functional [21,22,32] changes in the erythrocytes in individuals with C282Y/C282Y, which could lead to premature destruction. Scanning electron microscopy shows abnormal morphology with loss of the biconcave shape in some erythrocytes in individuals with C282Y/C282Y [20]. Functional studies have shown that erythrocytes in individuals with C282Y/C282Y are more rigid and less deformable compared to controls, making them more susceptible to shear stress [21,22,32].

Other studies have investigated the impact of elevated MCH, MCHC, or MCV, but not *HFE*, on HbA1c. A previous study using data from a laboratory information system of more than 400,000 outpatients with a first measurement of HbA1c demonstrated that the mean HbA1c level fell from 51 mmol/mol (6.8%) in the lowest (<27.5 pg) to 43 mmol/mol (6.0%) in the highest (>32.5 pg) MCH group [26]. Another study found that HbA1c is 5 mmol/mol lower in the high end compared to the low end of the reference range of MCHC [33]. Since intracellular erythrocyte glucose concentration equilibrates with plasma glucose via GLUT1 [34], the higher hemoglobin content per cell (reflected by increased MCHC) may dilute the relative concentration of intracellular glucose per hemoglobin molecule. This alteration in the intracellular glucose-to-hemoglobin ratio could reduce the extent of hemoglobin glycation with C282Y/C282Y and other *HFE* genotypes, contributing to the observed decrease in HbA1c level, as this reflects the proportion of glycosylated hemoglobin over the erythrocyte lifespan of 120 days.

This study was an observational cross-sectional population-based study, and while our mediation analyses suggested mechanisms, causality cannot be inferred. The prevalence of C282Y/C282Y was 0.32%, which was similar to another Danish study (0.36%) [5], slightly higher than in Sweden (0.23%) [6], and slightly lower than other populations of Northern European descent, such as in the USA and Canada (0.44%) [7], the UK (0.50%) [4], and Australia (0.60%) [35]. While there are other non-*HFE* hemochromatosis mutations, these are much rarer, and their clinical and laboratory phenotypes are very different from *HFE* hemochromatosis [8] and were therefore not included in this study. The unique aspect of the two included cohorts is that all participants were genotyped for C282Y and H63D and had their transferrin saturation levels measured, as opposed to routine clinical practice, which would be to genotype only those individuals with transferrin saturation levels above 45% [36].

We used the reticulocyte percentage as a proxy for the erythrocyte turnover, but this was not measured with biotin labeling, which is a more accurate measure; therefore, the exact values for erythrocyte survival should be interpreted with caution and are just relative indicators of decreased survival. The rate of production of erythrocytes with *HFE* genotypes is unknown but the reticulocyte hemoglobin was higher with C282Y/C282Y and other *HFE* genotypes in our study, indicating the later release of more mature reticulocytes from the bone marrow. The proportion mediated should be interpreted as a model-based estimate under the stated assumptions and is intended to provide a heuristic indication of relative contribution rather than an exact partitioning of causal effects.

We did not measure fasting glucose. Since the estimated average glucose (EAG) over the erythrocyte lifespan is calculated based on the HbA1c, which is always measured without fasting, the measured non-fasting venous plasma glucose had similar pre-analytical conditions than if we had measured the fasting glucose. Due to the glucose–HbA1c paradox, future studies should evaluate phlebotomy with serial HbA1c follow-up in prospective or interventional studies and alternative erythrocyte-independent glycemic biomarkers to HbA1c in individuals with C282Y/C282Y. Examples include the traditional and clinically approved 2 h oral glucose tolerance test (OGTT) and fasting glucose, which detect impaired glucose tolerance and diabetes [23], or the newer but not standardized biomarkers fructosamine (glycated serum proteins) [37] or glycated albumin [38], which reflect the average glucose over the last 2–3 weeks. Furthermore, it should be investigated if the decreased HbA1c causes a diagnostic delay of diabetes in individuals with C282Y/C282Y.

Conclusively, individuals with C282Y/C282Y have increased erythrocyte turnover (lower erythrocyte counts, and higher reticulocyte counts, reticulocyte percentage, and levels of bilirubin and LDH) and decreased HbA1c, which was partially mediated by increased transferrin saturation, MCHC, and MCV and decreased erythrocyte count. Despite decreased HbA1c, P-glucose was increased in individuals with C282Y/C282Y. Thus, HbA1c may not be a useful biomarker for diabetes diagnosis in individuals with C282Y/C282Y.

## 4. Materials and Methods

We included 130,779 participants from two Danish prospective studies. Individuals were randomly selected based on the Danish Civil Registration System Code to reflect the adult general population in two Danish regions. The Copenhagen General Population Study (CGPS, N = 109,574) included participants from 2003 to 2015 who were ≥20 years old [39]. The Danish General Suburban Population Study (GESUS, N = 21,205) was conducted 70 km south of Copenhagen and included participants from 2010 to 2013, who were ≥20 years old [40]. Participation was 45% in CGPS [41] and 43% in GESUS [40,42]. The studies collected similar information from questionnaires, physical examination, and blood samples. Individuals with the *HFE* genotype, blood counts, and of Danish descent were included. Participants were excluded due to missing *HFE* genotype, reticulocyte counts, or erythrocyte counts, or due to pregnancy, as these cause physiological changes in iron and glucose metabolism that may confound or distort the association between *HFE* variants, iron metabolism, and HbA1c (Supplementary Figure S1). In total, we included 103,734 participants from CGPS and 20,003 participants from GESUS, thus a total of 123,737 individuals. All participants provided written, informed consent. The studies were approved by Danish ethical committees (CGPS: H-KK-01-144/01 in 2008; GESUS: SJ-114, SJ-113 in 2009) and conformed to the principles of the Helsinki Declaration.

### 4.1. Questionnaire

In both CGPS and GESUS, information on menopause (women only), smoking (never, previous, and current), alcohol consumption (beer, wine, fortified wine, and liquor) in units per week (12 g/unit), recreational physical activity (none (<2 h/week), light (2–4 h/week), moderate (>4 h week at a steady pace), or strenuous (>4 h/week at a high intensity)), and prior infection within the last month (yes/no) were included.

### 4.2. Genetics

Direct genotyping for HFE genotypes C282Y (rs1800562) and H63D (rs1799945) was performed by a TaqMan assay (Applied Biosystems, Foster City, CA, USA) in CGPS and by KASPar allelic discrimination (LGC Genomics, Hoddesdon, UK) in GESUS [43]. Call rates were above 99% and were similar between methods. The C282Y genotypes did not differ from the Hardy–Weinberg equilibrium (*p* = 0.17), but H63D had a slight deviation (*p* = 0.048) (Supplementary Table S1). The following genotypes were investigated and coded as non-carrier/non-carrier (NC/NC = 1): H63D/NC (2), H63D/H63D (3), C282Y/NC (4), C282Y/H63D (5), and C282Y/C282Y (6), based on the pathogenicity of C282Y and the less pathogenic variant of H63D. It is well established that C282Y/C282Y disrupts the HFE protein, and this is called HFE hemochromatosis (or Type1A) when iron overload occurs and symptoms arise [44]. H63D is a modifier of heterozygous C282Y (compound heterozygosity), and this genotype is also associated with iron overload and hemochromatosis, although the penetrance is lower than for C282Y/C282Y [44,45]. There is more debate about the penetrance of hemochromatosis with C282Y/NC and H63D/H63D, but these variants confer mild iron overload, and the H63D/NC is generally considered a low-penetrance variant [35,44].

### 4.3. Laboratory Analyses

Non-fasting laboratory analyses were used. In CGPS, EDTA plasma transferrin and iron concentrations were measured by turbidimetry and absorption photometry, respectively, on Konelab autoanalyzer (Thermo Fisher Scientific, Vantaa, Finland), and EDTA plasma ferritin was measured by direct chemiluminometric assay on Advia Centaur (Siemens Healthineers, Erlangen, Germany). In GESUS, lithium heparin plasma transferrin, iron, and ferritin concentrations were measured by turbidimetry, colorimetry, and direct chemiluminometric assay on Cobas-6000 (Roche, Mannheim, Germany). Transferrin saturation (%) was calculated as iron concentration (µmol/L) divided by 2× transferrin concentration (µmol/L). Elevated transferrin saturation was defined as ≥45%.

Complete blood counts (CBCs) were measured on EDTA whole blood on ADVIA 120 Haematology System (Siemens Healthineers, Erlangen, Germany) in CGPS and on Sysmex XE-5000 (Sysmex Corp., Kobe, Japan) in GESUS. HbA1c was measured on EDTA whole blood using Tosoh G7 or G8 (Tosoh Corp., Tokyo, Japan) in GESUS. In GESUS, reticulocyte hemoglobin was only measured in 9195 individuals. In CGPS, reticulocytes and HbA1c were not measured, and ferritin was only measured in 8706 individuals (Supplementary Figure S1).

The following biochemical analyses were measured in venous plasma on Konelab autoanalyzer (CGPS) and Cobas-6000 (GESUS): glucose, alanine aminotransferase (ALAT), total bilirubin, and lactate dehydrogenase (LDH, only CGPS).

For all the above analyses, assay precision was tested daily, and assay accuracy was tested monthly using an external quality control program for all assays.

In GESUS, spot urine samples were collected at baseline on the same day as the blood collection and biobanked for 1–3 years at −80 °C before measurement. Oxidatively generated damage to DNA and RNA was quantified by measuring 8-oxo-7,8-dihydro-2′-deoxyguanosine (8-oxodG) and 8-oxo-7,8-dihydroguanosine (8-oxoGuo) adjusted for urinary creatinine concentration (unit: nmol/mmol creatinine) using ultra-performance LC-MS/MS on an Acquity UPL I-class system using a Xevo TQ-S triple quadrupole mass spectrometer (Waters Corp., Milford, MA, USA) [46]. The choice of LC-MS/MS was based on high specificity for these analytes and speed of analysis [47,48].

### 4.4. Diabetes

Individuals who affirmed their use of insulin or other antidiabetic medication in the questionnaire or who had a hospital-verified ICD code of diabetes mellitus (ICD-10: E10 or E11; ICD-8: 249 and 250) at baseline were classified as having known (prevalent) diabetes.

### 4.5. Estimated Erythrocyte Turnover

The erythrocyte turnover (%/day) was estimated from the erythrocyte (10^12^/L) and reticulocyte (10^9^/L) counts based on the assumption that the reticulocyte percentage (reticulocyte count/erythrocyte count) represents the production of erythrocytes per day in a steady state with constant production and removal of erythrocytes [25,49,50]. Thus, a reticulocyte fraction of 0.94% of all erythrocytes corresponds to an erythrocyte turnover of 0.94%/day and would represent 1/(0.0094/day) = 106 days of estimated erythrocyte survival.

### 4.6. Statistics

We used the statistical program STATA 14.2 (StataCorp., College Station, TX, USA: StataCorp LP). A *p*-value of less than 0.05 was considered statistically significant.

Descriptive tables are presented as raw values, with *p*-values (trend and by genotype) from linear regression or logistic regression adjusted for age, sex, and cohort.

Linear regression was used to estimate the association between biomarker level (erythrocyte count, reticulocyte count, estimated erythrocyte turnover, estimated erythrocyte survival, MCHC, reticulocyte hemoglobin, bilirubin, LDH, HbA1c, and p-glucose) and genotype status adjusting for sex, age (continuous), cohort, and blood donor status (ever/never). The difference in biomarker level for genotype compared to non-carrier is presented as the beta coefficient and standard error (SE). Adjusted means and 95% confidence intervals were obtained using post-estimation marginal predictions. We tested for interaction with sex.

As a sensitivity analysis, we performed a stepwise linear regression where genotype, age, sex, cohort, and blood donor status were entered a priori into the model. Stepwise selection was applied to: smoking (never, previous, or current), body mass index (continuous), alcohol consumption (gram/week), physical activity (none, light, moderate, or strenuous), and prior infection within the last month (yes/no). The covariates were chosen based on the consideration that these were not part of the direct pathway between genotype, iron overload, and biomarker level. The stepwise regression was first run against erythrocytes, and the same model was then used against all other outcomes. All the additional covariates were significant in the model and therefore included in all analyses of all outcomes.

In GESUS, we assessed if the association between *HFE* genotypes and HbA1c was indirectly mediated through transferrin saturation (<45% vs. ≥45%), MCHC, MCV, hsCRP, erythrocyte count, reticulocyte count, and blood donor status (ever/never) (N = 20,003). We also investigated indirect mediation by oxidative stress (by 8-oxoGuo or 8-oxodG adjusted for urinary creatinine) using the same model, but only in 3493 individuals. Specifically, we estimated the effect of *HFE* genotype (as a continuous variable, coded 1–6) on the mediator (the *a* path), the indirect effect of the mediator on HbA1c adjusted for genotype (the *b* path), and the direct effect of genotype on HbA1c conditional on the mediator (the *c* path). The indirect effect was defined as the product *a* × *b*, the direct effect as *c*, and the total effect as *c* + *a* × *b*. The percent mediated was calculated as (*a* × *b*)/(*c* + *a* × *b*). The mediation models were adjusted for age, sex, and blood donation. Models were estimated using structural equation modeling (*sem* command). Standard errors for the indirect and total effects were obtained using the delta method implemented via *nlcom*.

## Figures and Tables

**Table 1:** Characteristics of participants in the Copenhagen General Population Study (CGPS) and the Danish General Suburban Population Study (GESUS) combined.

	Total	NC/NC	H63D/NC	H63D/H63D	C282Y/NC	C282Y/H63D	C282Y/C282Y	*p* (Trend)
	N = 123,737	N = 81,397	N = 26,022	N = 2203	N = 11,722	N = 1994	N = 399	
GESUS	20,003	13,183 (65.9%)	4233 (21.2%)	351 (1.8%)	1857 (9.3%)	318 (1.6%)	61 (0.30%)	0.91
CGPS	103,734	68,214 (65.8%)	21,789 (21.0%)	1852 (1.8%)	9865 (9.5%)	1676 (1.6%)	338 (0.33%)	
Age, yr	57 (13)	57 (13)	57 (13)	57 (13)	57 (13)	57 (13)	56 (12)	0.073
Sex (M)	55,801 (45%)	36,723 (45%)	11,672 (45%)	1014 (46%)	5295 (45%)	910 (46%)	187 (47%)	0.83
Menopause	44,676 (66%)	29,351 (66%)	9494 (67%)	789 (67%)	4195 (66%)	704 (65%)	143 (67%)	0.72
Iron, µmol/L	14.3 (4.9)	13.8 (4.6)	14.9 (4.9) ***	16.9 (5.4) ***	15.3 (5.0) ***	18.6 (5.9) ***	25.5 (8.9) ***	<0.001
Transferrin, µmol/L	32.5 (5.7)	32.9 (5.7)	32.2 (5.6) ***	30.9 (5.4) ***	30.8 (5.2) ***	28.9 (4.9) ***	23.8 (4.7) ***	<0.001
TSAT, %	22.8 (8.8)	21.5 (7.9)	23.8 (8.6) ***	28.2 (10.3) ***	25.5 (9.2) ***	33.2 (11.8) ***	55.9 (22.0) ***	<0.001
Ferritin, µg/L	150.3 (185.0)	143.6 (144.5)	155.6 (260.4) ***	181.9 (186.0) ***	158.2 (146.9) ***	196.1 (188.5) ***	578.8 (924.6) ***	<0.001
Hematocrit, %	41.5 (3.4)	41.4 (3.4)	41.6 (3.4) ***	41.8 (3.3) ***	41.8 (3.4) ***	42.0 (3.5) ***	42.0 (3.4) **	<0.001
Hemoglobin, g/dL	14.1 (1.2)	14.1 (1.2)	14.1 (1.2) ***	14.3 (1.2) ***	14.2 (1.2) ***	14.4 (1.2) ***	14.4 (1.2) ***	<0.001
MCV, fl	90.2 (4.8)	90.0 (4.8)	90.5 (4.8) ***	91.3 (4.8) ***	90.8 (4.8) ***	91.8 (4.7) ***	93.2 (4.6) ***	<0.001
RDW, CV%	13.17 (1.02)	13.20 (1.03)	13.12 (1.00)***	13.02 (0.94)***	13.11 (1.06)***	12.94 (0.95)***	12.88 (0.94)***	<0.001
hsCRP, mg/L	2.5 (5.0)	2.5 (5.1)	2.5 (4.8)	2.6 (4.4)	2.5 (4.4)	2.4 (4.0)	3.1 (5.5) *	0.79
ALAT, IU/L	24.3 (16.9)	24.2 (16.3)	24.4 (19.1) ***	24.1 (15.2) ***	24.6 (16.3) ***	24.0 (14.2) ***	31.4 (23.8) ***	<0.001
8-oxoGuo (nmol/mmol creatinine)	2.4 (0.9)	2.4 (0.9)	2.4 (0.8)	2.5 (0.8)	2.5 (0.8) *	2.7 (1.1) ***	4.1 (2.8) ***	<0.001
8-oxodG (nmol/mmol creatinine)	1.9 (0.7)	1.8 (0.7)	1.8 (0.7)	2.0 (0.8)	1.9 (0.7)	1.9 (0.9)	2.0 (0.7)	0.019
BMI, kg/m^2^	26.2 (4.3)	26.2 (4.3)	26.2 (4.3)	26.3 (4.4)	26.2 (4.4)	26.0 (4.4) *	26.6 (4.8)	0.31
Smoking								0.13
Never	52,491 (42%)	34,521 (42%)	11,215 (43%)	917 (42%)	4804 (41%)	868 (44%)	166 (42%)	
Previous	49,662 (40%)	32,604 (40%)	10,413 (40%) *	901 (41%)	4813 (41%) **	782 (39%)	149 (37%)	
Current	21,584 (17%)	14,272 (18%)	4394 (17%)	385 (17%)	2105 (18%) *	344 (17%)	84 (21%)	
Alcohol, units/week ≤ 2	26,066 (21.9%)	17,285 (22.1%)	5411 (21.6%)	465 (22.0%)	2423 (21.5%)	398 (20.7%)	84 (22.1%)	0.02
Diabetes mellitus	5158 (4%)	3403 (4%)	1042 (4%)	101 (5%)	503 (4%)	88 (4%)	21 (5%)	0.29
Blood donor, ever	42,814 (35%)	28,085 (35%)	9004 (35%)	785 (36%)	4102 (35%)	697 (35%)	141 (35%)	0.20
Physical activity, none	7734 (6.3%)	5184 (6.4%)	1556 (6.0%)	128 (5.9%)	729 (6.3%)	116 (5.9%)	21 (5.3%)	0.07
Infection 1 month prior	4812 (3.9%)	3182 (3.9%)	1036 (4.0%)	64 (2.93%) *	438 (3.77%)	81 (4.10%)	11 (2.7%)	0.29

NC: non-carrier. * *p* < 0.05. ** *p* < 0.01. *** *p* < 0.001. *p*-values are based on linear regressions between genotype and continuous variables or logistic regressions between genotype and categorical variables adjusted for age and sex. In CGPS, ferritin was only measured in a subset of individuals. 8-oxoGuo and 8-oxo-dG were only measured in a subset of individuals and only in GESUS. Abbreviations: ALAT: Alanine aminotransferase; BMI: Body mass index; CGPS: Copenhagen General Population Study; GESUS: The Danish General Suburban Population Study; hsCRP: High-sensitivity C-reactive Protein; MCV: Mean Corpuscular Volume; RDW: Red Blood Cell Distribution Width (CV%: Coefficient of variation (%)); TSAT: Transferrin saturation; 8-oxoGuo: 8-oxo-7,8-dihydroguanosine; 8-oxodG: 8-oxo-7,8-dihydro-2′-deoxyguanosine.

## Data Availability

Due to ethical, legal, and privacy issues, the datasets used are only available upon request with approval from Danish research ethical committees and the steering committees for the cohorts. Requests to access the datasets should be directed to cel@regionsjaelland.dk for the GESUS cohort and boerge.nordestgaard@regionh.dk for the CGPS.

## Supplementary Materials

The following supporting information can be downloaded at: https://www.mdpi.com/article/10.3390/ijms27052410/s1.

## Abbreviations

The following abbreviations are used in this manuscript:

ALAT	Alanine aminotransferase
BMI	Body mass index
CGPS	Copenhagen General Population Study
GESUS	The Danish General Suburban Population Study
HbA1c	Hemoglobin A1c
hsCRP	High-sensitivity C-reactive Protein
LDH	Lactate dehydrogenase
MCHC	Mean Corpuscular Hemoglobin Concentration
MCH	Mean Corpuscular Hemoglobin
MCV	Mean Corpuscular Volume
RDW	Red Blood Cell Distribution Width (CV%: Coefficient of variation (%))
TSAT	Transferrin saturation
8-oxoGuo	8-oxo-7,8-dihydroguanosine
8-oxodG	8-oxo-7,8-dihydro-2′-deoxyguanosine
