# Supplementary material for: C282Y Homozygosity Increases Erythrocyte Turnover and Decreases HbA1c—A Population-Based Study
Source: Int J Mol Sci. 2026 Mar 5;27(5):2410. doi: 10.3390/ijms27052410 (PMC12986277; doi:10.3390/ijms27052410)
Supplement: Supplementary file 1 [file ijms-27-02410-s001.zip › ijms-4123615-supplementary.pdf]

Supplementary file for:

Article

C282Y homozygosity increases erythrocyte turnover and decreases HbA1c - A population-based study

Rebekka Hillingsø, Alisa Devedzic Kjaergaard, Morten Kranker Larsen, Thomas Mandrup-Poulsen, Henrik Enghusen Poulsen, Mathis Mottelson, Jesper Brix Petersen, Børge Grønne Nordestgaard, Hans Carl Hasselbalch, Stig Egil Bojesen, Jens Helby, Andreas Glenthøj, Christina Ellervik

Supplementary Figure S1. Flowchart of Danish population-based cohorts.

CGPS: Copenhagen General Population Study. GESUS: The Danish General Suburban Population Study.

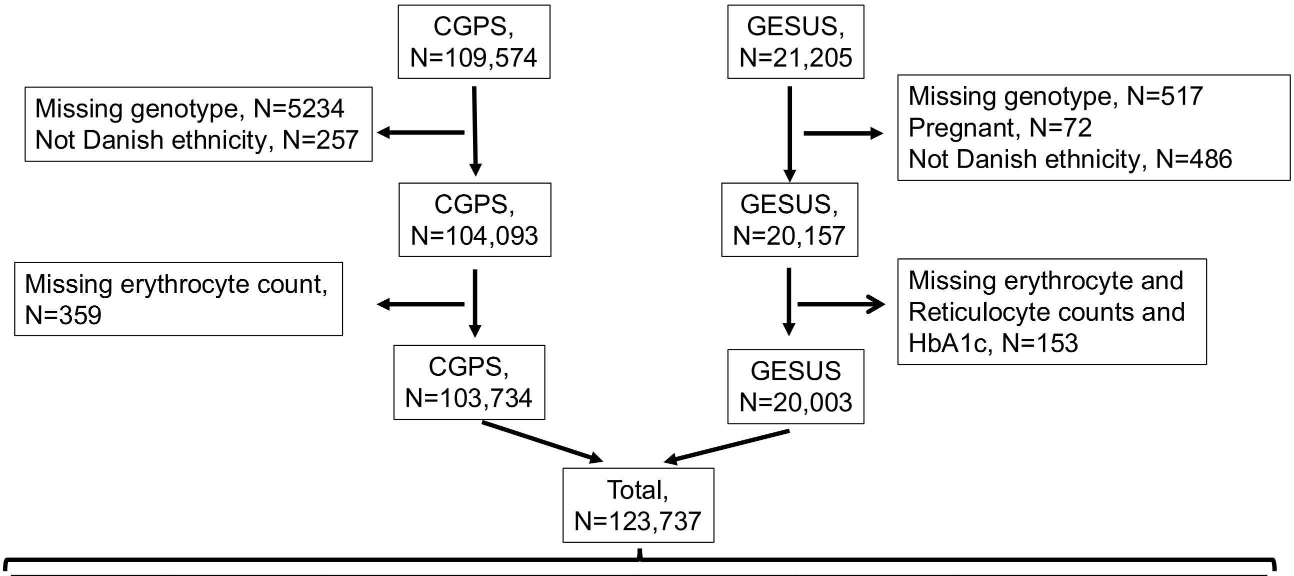

N	*HFE* Genotype	TSAT	Ferritin	8-oxoGuo 8-oxodG	Erythrocytes	Reticulocytes	HbA1c
CGPS	103,734	103,341	8706	-	103,734	-	-
GESUS	20,003	19,991	20,000	3493	20,003	20,003	20,003

Supplementary Table S1. Hardy-Weinberg Equilibrium for C282Y and H63D in CGPS and GESUS combined

	Observed			Expected	
C282Y	N	%		N	%
GG	109,622	88.59		109,649	88.61
GA	13,716	11.08		13,663	11.04
AA	399	0.32		426	0.34
Total	123,737			123,738	
Allele frequency					
G	0.941				
A	0.059				
X^2	1.88				
df	1				
p	0.17				
	Observed			Expected	
H63D	N	%		N	%
CC	93,518	75.58		93,439	75.51
GC	28,016	22.64		28,174	22.77
GG	2,203	1.78		2,124	1.72
Total	123,737			123,737	
Allele frequency					
C	0.869				
G	0.131				
X^2	3.91				
df	1				
p	0.048				

Supplementary Table S2. Characteristics of participants in the Copenhagen General Population Study (CGPS)

NC: non-carrier. * p<0.05. ** p<0.01. *** p<0.001. p-values are based on linear regressions between genotype and continuous variables or logistic regressions between genotype and categorical variables adjusted for age and sex. Ferritin was only measured in a subset of individuals.

	Total	NC/NC	H63D/NC	H63D/H63D	C282Y/NC	C282Y/H63D	C282Y/C282Y	p-value
	N=103,734	N=68,214	N=21,789	N=1,852	N=9,865	N=1,676	N=338	
Age, yr	57 (13)	57 (13)	57 (13)	57 (13)	57 (13)	57 (13)	56 (12)	0.20
Sex (M)	46,659 (45%)	30,685 (45%)	9,744 (45%)	833 (45%)	4,460 (45%)	773 (46%)	164 (49%)	0.64
Menopause	37,785 (67%)	24,823 (67%)	8,024 (67%)***	679 (67%)***	3,550 (66%)***	590 (66%)***	119 (68%)***	0.74
Iron, µmol/l	14.5 (4.9)	13.9 (4.6)	15.0 (4.9) ***	17.0 (5.5) ***	15.4 (5.0) ***	18.7 (5.9) ***	25.7 (8.6) ***	<0.001
Transferrin, µmol/l	32.3 (5.8)	32.8 (5.8)	32.1 (5.7) ***	30.9 (5.6) ***	30.7 (5.3) ***	28.8 (5.0) ***	23.6 (4.8) ***	<0.001
Transferrin saturation, %	23.1 (9.0)	21.8 (8.0)	24.1 (8.7)	28.5 (10.5)	25.8 (9.4)	33.6 (12.0)	57.1 (22.1)	<0.001
Ferritin, µg/L	133.5 (226.6)	126.2 (124.1)	144.2 (424.9) **	155.8 (121.1)	139.1 (124.3)	161.8 (157.4)	614.9 (820.3) ***	<0.001
Hematocrit, %	41.3 (3.4)	41.2 (3.4)	41.4 (3.4) ***	41.6 (3.3) ***	41.5 (3.4) ***	41.8 (3.4) ***	41.9 (3.4) ***	<0.001
Hemoglobin, g/dL	14.1 (1.2)	14.1 (1.2)	14.2 (1.2) ***	14.3 (1.2) ***	14.2 (1.2) ***	14.4 (1.2) ***	14.5 (1.2) ***	<0.001
MCV, fl	89.8 (4.8)	89.6 (4.8)	90.1 (4.7) ***	90.9 (4.8) ***	90.4 (4.7) ***	91.4 (4.5) ***	92.7 (4.3) ***	<0.001
RDW, CV%	13.11 (0.83)	13.15 (0.83)	13.07 (0.81)***	12.97 (0.77)***	13.05 (0.89)***	12.91 (0.81)***	12.85 (0.73)***	<0.001
hsCRP, mg/l	2.4 (4.9)	2.5 (5.0)	2.4 (4.8)	2.5 (4.4)	2.4 (4.3)	2.4 (4.0)	2.8 (4.2)	0.52
ALAT, IU/L	23.7 (16.3)	23.7 (15.4)	23.8 (19.6)	23.4 (15.0)	24.0 (15.3)	23.4 (13.8)	31.1 (23.1) ***	0.004
BMI, kg/m2	26.1 (4.3)	26.2 (4.3)	26.1 (4.3)	26.2 (4.2)	26.1 (4.3)	25.9 (4.3)*	26.6 (4.6)*	0.20
Smoking								0.10
Never	43,743 (42%)	28,777 (42%)	9,340 (43%)	759 (41%)	3,999 (41%)	724 (43%)	144 (43%)	
Previous	41,976 (40%)	27,537 (40%)	8,787 (40%)	766 (41%)	4,096 (42%)*	663 (40%)	127 (38%)	
Current	18,015 (17%)	11,900 (17%)	3,662 (17%)	327 (18%)	1,770 (18%)**	289 (17%)	67 (20%)	
Alcohol, units/week≤2	18,305 (19%)	12,135 (18%)	3,784 (18%)	337 (19%)	1,728 (18%)	263 (16%)*	58 (18%)	0.05
Diabetes mellitus	4,193 (4%)	2,774 (4%)	856 (4%)	78 (4%)	395 (4%)	71 (4%)	19 (6%)	0.73
Blood donor, ever	36,322 (35%)	23,831 (35%)	7,588 (35%)	665 (36%)	3,527 (36%)	593 (35%)	118 (35%)	0.16
Physical activity, none	6,472 (6.3%)	4,335 (6.4%)	1,312 (6.1%)	106 (5.8%)	602 (6.2%)	99 (6.0%)	18 (5.3%)	0.07
Infection 1 month prior	4,090 (4.0%)	2,708 (4.0%)	873 (4.0%)	57 (3.1%)	376 (3.8%)	65 (3.9%)	11 (3.3%)	0.31

Supplementary Table S3. Characteristics of participants in the Danish General Suburban Population Study (GESUS)

NC: non-carrier. * p<0.05. ** p<0.01. *** p<0.001. p-values are based on linear regressions between genotype and continuous variables or logistic regressions between genotype and categorical variables adjusted for age and sex. 8-oxoGuo and 8-oxo-dG were only measured in a subset of individuals.

	Total	NC/NC	H63D/NC	H63D/H63D	C282Y/NC	C282Y/H63D	C282Y/C282Y	p-value
	N=20,003	N=13,183	N=4,233	N=351	N=1,857	N=318	N=61	
Age, yr	56 (14)	56 (14)	56 (13)	56 (14)	56 (14)	55 (14)	54 (12)	0.62
Sex (M)	9,142 (46%)	6,038 (46%)	1,928 (46%)	181 (52%)	835 (45%)	137 (43%)	23 (38%)	0.17
Menopause	6,891 (63%)	4,528 (63%)	1,470 (64%)	110 (65%)	645 (63%)	114 (63%)	24 (63%)	1.00
Iron, µmol/l	13.7 (4.9)	13.1 (4.6)	14.2 (5.0) ***	16.3 (5.2) ***	14.5 (5.0) ***	18.1 (5.9) ***	24.1 (10.0) ***	<0.001
Transferrin, µmol/l	33.1 (5.0)	33.5 (5.0)	33.0 (4.9) ***	30.9 (4.2) ***	31.4 (4.7) ***	29.6 (4.4) ***	25.1 (4.3) ***	<0.001
Transferrin saturation, %	21.1 (7.9)	19.9 (7.1)	22.0 (7.7) ***	26.8 (8.8) ***	23.4 (8.2) ***	31.1 (10.6) ***	49.4 (20.6) ***	<0.001
Ferritin, µg/L	157.6 (163.2)	151.2 (152.0)	160.4 (143.5) ***	193.9 (208.2) ***	167.0 (155.4) ***	211.1 (199.0) ***	565.8 (965.5) ***	<0.001
Hematocrit, %	42.8 (3.3)	42.7 (3.3)	42.9 (3.3) ***	43.1 (3.2)	43.0 (3.3) ***	43.0 (3.6) *	42.1 (3.4)	<0.001
Hemoglobin, g/dL	14.0 (1.3)	14.0 (1.2)	14.1 (1.4) ***	14.2 (1.2) **	14.1 (1.2) ***	14.2 (1.2) ***	13.9 (1.2)	<0.001
MCV, fl	92.4 (4.5)	92.2 (4.5)	92.6 (4.5) ***	93.5 (4.2) ***	92.9 (4.6) ***	94.0 (4.8) ***	96.1 (5.4) ***	<0.001
RDW, CV%	13.45 (1.68)	13.49 (1.70)	13.38 (1.65)***	13.32 (1.54)	13.40 (1.65)*	13.13 (1.49)***	13.00 (1.67)*	<0.001
hsCRP, mg/l	2.8 (5.5)	2.7 (5.7)	2.7 (5.2)	3.0 (4.5)	2.7 (4.9)	2.7 (4.1)	4.2 (10.0)*	0.46
ALAT, IU/L	27.0 (19.3)	26.8 (20.2)	27.3 (16.5)	27.5 (15.4)	27.7 (20.2)	27.1 (15.5)	32.9 (27.5)**	0.010
8-oxoGuo (nmol/mmol creatinine)	2.4 (0.9)	2.4 (0.9)	2.4 (0.8)	2.5 (0.8)	2.5 (0.8)*	2.7 (1.1)***	4.1 (2.8)***	<0.001
8-oxodG (nmol/mmol creatinine)	1.9 (0.7)	1.8 (0.7)	1.8 (0.7)	2.0 (0.8)	1.9 (0.7)	1.9 (0.9)	2.0 (0.7)	0.019
BMI, kg/m2	26.7 (4.7)	26.7 (4.6)	26.8 (4.7)	27.0 (4.9)	26.7 (4.6)	26.4 (4.6)	26.6 (5.5)	0.68
Smoking								0.94
Never	8,748 (44%)	5,744 (44%)	1,875 (44%)	158 (45%)	805 (43%)	144 (45%)	22 (36%)	
Previous	7,686 (38%)	5,067 (38%)	1,626 (38%)	135 (38%)	717 (39%)	119 (37%)	22 (36%)	
Current	3,569 (18%)	2,372 (18%)	732 (17%)	58 (17%)	335 (18%)	55 (17%)	17 (28%)*	
Alcohol, units/week≤2	7,761 (39%)	5,150 (39%)	1,627 (38%)	128 (36.5%)	695 (37%)	135 (43%)	26 (43%)	0.26
Diabetes mellitus	965 (5%)	629 (5%)	186 (4%)	23 (7%)	108 (6%)*	17 (5%)	2 (3%)	0.13
Blood donor, ever	6,492 (32%)	4,254 (32%)	1,416 (33%)	120 (34%)	575 (31%)	104 (33%)	23 (38%)	0.91
Physical activity, none	1,262 (6.5%)	849 (6.6%)	244 (5.9%)	22 (6.5%)	127 (7.0%)	17 (5.6%)	3 (4.9%)	0.79
Infection 1 month prior	722 (3.7%)	474 (3.6%)	163 (3.9%)	7 (2.0%)	62 (3.4%)	16 (5.11%)	0	0.73

Supplementary Table S4. Association between HFE genotype and outcome in adjusted linear regression analyses.

Analyses are adjusted for sex, age, cohort blood donor status, smoking, body mass index, alcohol consumption, physical activity, BMI, and infection.

Outcome	Genotype	Difference	SE	Mean	SE
Erythrocytes (10^{12}/L)	Non-carriers	0.000	0.000	4.613	0.001
Erythrocytes (10^{12}/L)	H63D/NC	-0.003	0.003	4.610	0.002
Erythrocytes (10^{12}/L)	H63D/H63D	-0.029	0.008	4.584	0.008
Erythrocytes (10^{12}/L)	C282Y/NC	-0.007	0.004	4.606	0.003
Erythrocytes (10^{12}/L)	C282Y/H63D	-0.030	0.008	4.583	0.008
Erythrocytes (10^{12}/L)	C282Y/C282Y	-0.126	0.018	4.486	0.018
Reticulocytes (10^{9}/L)	Non-carriers			49.151	0.130
Reticulocytes (10^{9}/L)	H63D/NC	1.387	0.264	50.538	0.230
Reticulocytes (10^{9}/L)	H63D/H63D	1.703	0.806	50.854	0.795
Reticulocytes (10^{9}/L)	C282Y/NC	1.294	0.371	50.445	0.348
Reticulocytes (10^{9}/L)	C282Y/H63D	2.982	0.852	52.133	0.842
Reticulocytes (10^{9}/L)	C282Y/C282Y	5.314	1.876	54.465	1.871
HbA1c (mmol/mol)	Non-carriers			37.838	0.048
HbA1c (mmol/mol)	H63D/NC	-0.381	0.098	37.458	0.085
HbA1c (mmol/mol)	H63D/H63D	-0.651	0.298	37.187	0.294
HbA1c (mmol/mol)	C282Y/NC	-0.304	0.137	37.535	0.129
HbA1c (mmol/mol)	C282Y/H63D	-0.974	0.316	36.864	0.312
HbA1c (mmol/mol)	C282Y/C282Y	-1.939	0.695	35.899	0.693
P-Glucose (mmol/L)	Non-carriers			5.397	0.004
P-Glucose (mmol/L)	H63D/NC	0.001	0.009	5.398	0.007
P-Glucose (mmol/L)	H63D/H63D	-0.001	0.026	5.396	0.026
P-Glucose (mmol/L)	C282Y/NC	0.003	0.012	5.400	0.011
P-Glucose (mmol/L)	C282Y/H63D	0.030	0.027	5.427	0.027
P-Glucose (mmol/L)	C282Y/C282Y	0.143	0.060	5.540	0.060
eAG (mmol/L)	Non-carriers			6.317	0.007
eAG (mmol/L)	H63D/NC	-0.055	0.014	6.262	0.012
eAG (mmol/L)	H63D/H63D	-0.095	0.043	6.223	0.043
eAG (mmol/L)	C282Y/NC	-0.044	0.020	6.273	0.019
eAG (mmol/L)	C282Y/H63D	-0.142	0.046	6.176	0.045
eAG (mmol/L)	C282Y/C282Y	-0.282	0.101	6.035	0.101
Bilirubin (mmol/L)	Non-carriers			10.548	0.018
Bilirubin (mmol/L)	H63D/NC	0.145	0.036	10.693	0.031
Bilirubin (mmol/L)	H63D/H63D	0.407	0.110	10.955	0.108
Bilirubin (mmol/L)	C282Y/NC	0.163	0.050	10.711	0.047
Bilirubin (mmol/L)	C282Y/H63D	0.366	0.115	10.914	0.113
Bilirubin (mmol/L)	C282Y/C282Y	1.461	0.255	12.009	0.254
LDH (mmol/L)	Non-carriers			157.146	0.129

LDH (mmol/L)	H63D/NC	1.157	0.261	158.303	0.227
LDH (mmol/L)	H63D/H63D	3.200	0.794	160.346	0.784
LDH (mmol/L)	C282Y/NC	0.720	0.362	157.866	0.338
LDH (mmol/L)	C282Y/H63D	2.568	0.828	159.714	0.818
LDH (mmol/L)	C282Y/C282Y	4.672	1.835	161.818	1.831
Erythrocyte turnover (%/d)	Non-carriers			1.057	0.003
Erythrocyte turnover (%/d)	H63D/NC	0.030	0.006	1.087	0.005
Erythrocyte turnover (%/d)	H63D/H63D	0.051	0.017	1.109	0.017
Erythrocyte turnover (%/d)	C282Y/NC	0.029	0.008	1.086	0.007
Erythrocyte turnover (%/d)	C282Y/H63D	0.077	0.018	1.135	0.018
Erythrocyte turnover (%/d)	C282Y/C282Y	0.187	0.040	1.244	0.040
Erythrocyte survival (days)	Non-carriers			103.353	0.264
Erythrocyte survival (days)	H63D/NC	-2.413	0.537	100.939	0.468
Erythrocyte survival (days)	H63D/H63D	-4.116	1.641	99.236	1.620
Erythrocyte survival (days)	C282Y/NC	-1.869	0.756	101.484	0.708
Erythrocyte survival (days)	C282Y/H63D	-5.956	1.737	97.397	1.716
Erythrocyte survival (days)	C282Y/C282Y	-13.241	3.822	90.112	3.813
MCH (pg)	Non-carriers			30.530	0.006
MCH (pg)	H63D/NC	0.242	0.012	30.772	0.010
MCH (pg)	H63D/H63D	0.652	0.035	31.182	0.035
MCH (pg)	C282Y/NC	0.377	0.016	30.907	0.015
MCH (pg)	C282Y/H63D	0.899	0.037	31.429	0.036
MCH (pg)	C282Y/C282Y	1.607	0.082	32.137	0.082
MCHC (g/L)	Non-carriers			339.608	0.046
MCHC (g/L)	H63D/NC	0.789	0.094	340.397	0.082
MCHC (g/L)	H63D/H63D	2.191	0.287	341.799	0.283
MCHC (g/L)	C282Y/NC	1.121	0.131	340.729	0.123
MCHC (g/L)	C282Y/H63D	2.975	0.300	342.583	0.296
MCHC (g/L)	C282Y/C282Y	4.389	0.666	343.997	0.664
Reticulocyte hemoglobin (pg)	Non-carriers			33.914	0.061
Reticulocyte hemoglobin (pg)	H63D/NC	0.127	0.125	34.041	0.109
Reticulocyte hemoglobin (pg)	H63D/H63D	0.654	0.385	34.568	0.380
Reticulocyte hemoglobin (pg)	C282Y/NC	0.295	0.173	34.209	0.162
Reticulocyte hemoglobin (pg)	C282Y/H63D	0.973	0.390	34.887	0.385
Reticulocyte hemoglobin (pg)	C282Y/C282Y	1.751	0.899	35.665	0.897
